# Building a public health network for prevention of suicides in Macedonia in accordance to the German model - “Nuernberg Alliance Against Depression”: “Macedonian Alliance Against Depression”- MAAD

**DOI:** 10.1186/1878-5085-5-S1-A93

**Published:** 2014-02-11

**Authors:** Kneginja Richter, Mirjana Polazarevska, Günter Niklewski

**Affiliations:** 1 Clinic for Psychiatry and Psychotherapy, Nuremberg, Germany; 2 Georg Simon Ohm Technical University for applied sciences, Nuremberg, Germany; 3 Medical Faculty, University Goce Delcev, Stip, Macedonia, FYROM; 4 Clinic for Psychiatry, Medical Faculty, University Cyril and Methodus, Skopje, Macedonia, FYROM

## Goal of the project

The main goal of the program is prevention of suicide through early prediction and detection of depression. For the sustainability of the program a national centre for prevention and treatment of depression and suicide shell be established in the country. The Macedonian alliance against depression should be a network of experts with the aim to promote the healthcare of depressed patients and to reduce the number of suicide and suicide attempts in Macedonia.

## Background

Every year almost one million people die from suicide; a global mortality rate of 16 per 100,000, or one death every 40 seconds [1]. In the last 45 years suicide rates have increased by 60% worldwide. Suicide is among the three leading causes of death among people aged 15 - 44 years in some countries, and the second leading cause of death in the 10-24 years age group [1].

A major proportion of the suicides are committed in the context of depression. Obviously, among the group of general practitioners there is a deficit in the recognition and therapy of depressive syndromes [2]. Therefore the importance of primary care for an efficient suicide prevention program is evident. This was already shown in the Gotland Study [2]. In this study an educational programme for general practitioners was carried out to improve the diagnosis and treatment of depressive disorders and suicidality. The evaluation showed an impressive decrease (nearly 50 %) of suicides.

The Rate of Suicides in Year 2010 was 6, 7 for Macedonia and 9, 9 for Germany, per 100.000 inhabitants. The epidemiological data on suicide attempts during a 10-year-period (1999-2008) based on 1683 participants who attempted suicides in the Region of Skopje, Macedonia showed a stable trend over the last decade. It has also shown female predominance of suicide attempts with a greater number of attempts during the summer months [3]. During 2001/ 2002 an intervention programme on four intervention levels has been conducted in the city of Nuremberg, Germany („Nuremberg Alliance Against Depression“), to improve the care for individuals suffering from depressive disorders. The project was funded by the German Federal Ministry for Education and Research in the framework of the German Research Network on Depression and Suicidality. The “Nuremberg Alliance Against Depression” leads to a decrease in overall suicidality (suicides and suicide attempts) of more than 20 % in Nuremberg and the region. Meanwhile 14 regions in Germany initiated similar projects under the common roof of the „Alliance Against Depression“, another 30 – 40 other German regions are in an early stage of preparation [4]. Since 2001, there is an active cooperation between Nuremberg (Germany) and Skopje (Macedonia) supported by WHO, Municipalities, Ministry of Health and two biggest state Universities in Macedonia. The well established cooperation can be used as a tool for further research projects [5].
